# Improving optimal prompt learning through multilayer fusion and latent dirichlet allocation

**DOI:** 10.3389/frobt.2025.1579990

**Published:** 2025-06-06

**Authors:** Qinghua Chen, Jessica Korneder, Osamah A. Rawashdeh, Yanfeng Wang, Wing-Yue Geoffrey Louie

**Affiliations:** ^1^ Intelligent Robotics Laboratory, Oakland University, Rochester, MI, United States; ^2^ Embedded Systems Research Lab, Oakland University, Rochester, MI, United States; ^3^ College of Electrical and Information Engineering, Zhengzhou University of Light Industry, Zhengzhou, Henan, China; ^4^ Applied Behavior Analysis Clinic, Oakland University, Rochester, MI, United States

**Keywords:** few-shot prompt learning, multilayer fusion, LDA topic integration, human-robot interaction, extracting valuable information

## Abstract

Recent advances in few-shot learning have demonstrated the potential of prompt-based techniques with pre-trained models, eliminating the need for extensive fine-tuning. However, challenges such as obtaining optimal prompts and addressing data scarcity in specialized domains remain challenging. We introduce a novel framework incorporating a Global Attention Mechanism (GAM) that effectively integrates features from multiple layers of pre-trained language models, enhanced by Latent Dirichlet Allocation (LDA) generated topic features for prompt optimization. Extensive experiments on four datasets consistently show that our approach outperforms state of-the-art baselines. The strategic integration of GAM with layer-specific features and LDA topics proves particularly effective in extracting valuable latent information for few-shot learning scenarios, yielding significant improvements in specialized domains, as evidenced by enhanced performance in therapeutic dialogue classification within a Applied Behavior Analysis clinical dataset.

## 1 Introduction

Human-robot interaction (HRI) has been transformed by natural language processing (NLP) technologies, enabling robots to comprehend complex linguistic inputs and expanding their applications across industrial, healthcare, and educational settings Atuhurra (2024); Koubaa (2023). However, a critical challenge persists: acquiring sufficient labeled demonstration data for model training, particularly in few-shot learning scenarios where data availability is limited Hejna III and Sadigh (2023). This challenge is exacerbated in real-world HRI applications, especially within sensitive domains like healthcare and education, where data collection faces significant privacy constraints, high costs, and ethical complexities Chen et al. (2022).

Current few-shot learning techniques largely rely on pre-training models with large-scale multitask datasets, followed by fine-tuning on smaller, domain-specific datasets. Although effective, fine-tuning these large models is complex and requires specialized expertise Bragg et al. (2021); Liu et al. (2024). Recently, the use of specific prompts or instructions to guide large language models (LLMs) has significantly enhanced their ability to perform more intricate natural language processing (NLP) tasks. Prompt learning, which focuses on the tuning of task-specific parameters of pre-trained language models (PLM), has shown considerable success, often outperforming traditional fine-tuning on low-resource datasets Liu et al. (2024); Zhou et al. (2022). These advances in prompt learning present compelling new opportunities for enhancing HRI capabilities.

State-of-the-art prompt-based methods have demonstrated effectiveness through various approaches: incorporating labeled human feedback in prompt generation Bai et al. (2022); Zhou et al. (2023), manually adjusting task-specific parameters Rajeswaran et al. (2017), and employing performance enhancement techniques to learn from high-quality demonstrations Wang et al. (2023) and extract insights from ambiguous training data Zha et al. (2021). However, adapting these techniques to HRI scenarios presents significant challenges. The creation of high-quality prompts requires substantial domain expertise and time investment Koubaa (2023), while the inherently limited size of HRI datasets restricts their coverage of the overall data distribution, resulting in pre-training corpora that inadequately represent the diversity of environments and tasks Xu et al. (2022). As a result, prompt-based models often fail to capture sparse but essential information within limited demonstration data, especially for high-level, abstract, or ambiguous tasks, significantly compromising prompt effectiveness in downstream applications Gu et al. (2022).

Given the inherent limitations of current methods, especially in the HRI domain with scarce demonstration samples, it is essential to explore approaches that effectively extract additional information from limited demonstrations. To address this challenge, we propose a novel methodology that enhances both prompt generation and model performance. Our framework leverages multi-layer feature vectors from pre-trained language models to capture information that may have been overlooked during prompt generation, while simultaneously incorporating external latent features through the topic keywords derived from third-party models. Extensive experimental validation demonstrates the efficacy of our approach across multiple datasets, particularly in real-world HRI scenarios with limited data availability.

Our key contributions include:

• A Global Attention Mechanism that enhances prompt learning by integrating feature representations across multiple layers of pre-trained language models.

• A novel integration method incorporating Latent Dirichlet allocation topic features from external models to enrich prompt generation.

• Comprehensive empirical validation across multiple datasets, encompassing sentiment analysis tasks and real-world HRI scenarios with constrained data availability.

## 2 Related work

This section explores approaches to prompt optimization through two key aspects. First, we examine model architectures that employ either direct language models (LM) for prompt generation or combine reinforcement learning with language models (LM-RL). Second, we investigate methods for maximizing prompt optimization with limited data by extracting valuable information from the different layers of language model BERT and incorporating supplementary information from external models.

### 2.1 Prompt optimization of few-shot learning

Current prompt optimization methods focus mainly on enhancing the model and fully exploiting the available data. Architectural approaches like PTuning v2 Liu et al. (2021), Google’s instruction tuning Wei et al. (2022), and Prompt-DT Xu et al. (2022) optimize model structures for prompt generation. Alternative methods, including APE Zhou et al. (2023), OPRO Yang et al. (2024), and PromptBreeder Fernando et al. (2024), utilize larger models from the PaLM2 Anil et al. (2023) and GPT model families to propose and validate prompt candidates. Although AutoPrompt Shin et al. (2020) employs gradient-based search for prompt editing, it requires model gradient access. These LLM-based approaches face challenges in interpretability, cross-model reusability, particularly due to their dependence on fine-tuning dataset size Wei et al. (2022). This limitation restricts their application in scenarios like HRI, where the demonstration data is limited.

Compared to typical prompt-tuning approaches, the pre-trained language model and reinforcement learning (LM-RL) frameworks offer an alternative by optimizing prompts without expensive gradient computations. Recent applications Hao et al. (2023) include aesthetic optimization in text-to-image generation tasks and Prompt-OIRL Sun et al. (2023) that utilize offline inverse reinforcement learning to optimize query-prompt pairs. However, the approach requires substantial manually labeled rewards for training its proxy reward model, which introduces additional resource overhead and data dependencies. On the other hand, merging LM-RL frameworks show promise in prompt optimization. In Prewrite Kong et al. (2024), two prompt-rewriting LLMs are trained using reinforcement learning to optimize performance on a given downstream task based on large models. RLPROMPT Deng et al. (2022) and TEMPERA Zhang et al. (2023) propose prompt optimization approaches with pre-trained LM and reinforcement learning (LM-RL) based on relatively smaller language models. In particular, the LM-RL architecture demonstrates robust performance regardless of prompt pool size or few-shot example quantity, making it particularly suitable for data-constrained scenarios.

In addition to model development, some studies have shown that incorporating human feedback or expert demonstrations into training optimization prompts can improve performance Bai et al. (2022); Jung and Kim (2024); Zhou et al. (2023). These approaches face significant limitations, including high resource requirements for labeled feedback and demonstrations, substantial computational and financial costs, and limited effectiveness in small datasets where biases are more pronounced.

Beyond leveraging LM-RL model structures for optimal prompt generation, a critical challenge in few-shot learning lies in maximizing information extraction from limited available data. This requires innovative approaches to uncover crucial and latent information that might otherwise be overlooked. The efficient integration of such information into the prompt generation process represents a promising research direction to improve the performance of few-shot learning.

### 2.2 Leveraging different layers of LM and supplementary information to LM

Manually selecting parameters Zhang N. et al. (2022) and incorporating processed demonstrations Tang et al. (2024), can result in loss of vital information due to the need for manual parameter design, particularly when working with limited data. The internals of the language model offer an alternative source of valuable information. Research shows that the bidirectional encoder representations from transformers (BERT) layers possess distinct specializations Wolf (2019); de Vries et al. (2020), and the earlier layers often contain crucial information. Studies suggest incorporating fluency-related information into the LM, which includes sparse features or abstract information that cannot be easily extracted from the last layer of the LM Zhang N. et al. (2022). Various approaches have demonstrated the benefits of the performance boost from multi-layer integration, rather than using only the BERT last layer Zhang et al. (2021). The dynamic fusion mechanism on the encoder and the knowledge distillation paradigm on the decoder attention Vaswani et al. (2017) provide rich information for the model by integrating the multilayer representations of BERT. These lower layers contain fluency-related features and abstract information that are not readily available in the final layer. Researchers suggest that selectively adding prompts to specific layers of the model is more effective than applying prompts to every layer Jia et al. (2022). Liu et al. (2021) highlights the importance of focusing on key layers for prompt optimization with LMs. However, indiscriminate combination of features from all layers may introduce redundancy and noise, particularly in small dataset scenarios where lower layers might contain overlooked but valuable information. Our method explores this approach to enhance prompt optimization.

Alternative approaches explore LM fine-tuning with supplementary models. For example, Venugopalan and Gupta (2022) uses minimal aspect seed words from each aspect category to guide the model, which is combined with BERT-based semantic similarity. Peinelt et al. (2020) demonstrates that topic-informed BERT (tBERT) achieves improvements in multiple semantic similarity prediction datasets. Furthermore, Xiang et al. (2023) proposes bidirectional encoder representations from transformers-latent dirichlet allocation (BERT-LDA) in the context of online health communities, achieving more accurate topic identification and sentiment analysis. Zhang P. et al. (2022) proposes a method that combines weighted latent dirichlet allocation (LDA), Word2Vec, and BERT vectors for text classification. Given the role of supplementary models, our research explores efficient ways to incorporate supplementary models for feature extraction within the LM-RL framework, specifically targeting improved prompt learning performance in small dataset scenarios.

In this paper, we extend the LM-RL framework by addressing information loss inherent in final-layer-only approaches. Our approach leverages feature representations from multiple layers of the language model and implements a Global Attention Mechanism to effectively synthesize cross-layer information. Additionally, we incorporate a third-party model to provide complementary implicit features, expanding the set of available features for optimization. This comprehensive approach enhances the information capture capabilities of the LM-RL network. We validate our framework through extensive experiments on three few-shot learning classification tasks, and we explored its applicability in a specific HRI scenario involving autism patient treatment, where the demonstrations are limited, the situation is more complex and lacks the stable distribution present in the classification tasks of the other three datasets.

## 3 Methods

To enhance prompt learning by effectively extracting additional information, this paper builds upon the LM-RL framework and addresses a key challenge: some essential information is overshadowed when relying solely on features from the final layer of a language model (LM) as input for reinforcement learning (RL). To tackle this, we explore various methods for integrating features from different layers of the LM, incorporating supplementary implicit information to optimize prompt generation. The proposed approach is detailed across three main subsections: Section 3.1 introduces the foundational LM-RL framework; Section 3.2 describes the fusion of multi-layer features from the LM; and Section 3.3 explores the integration of Latent Dirichlet allocation (LDA) with LM-derived representations.

### 3.1 Basic LM-RL framework

**FIGURE 1:**
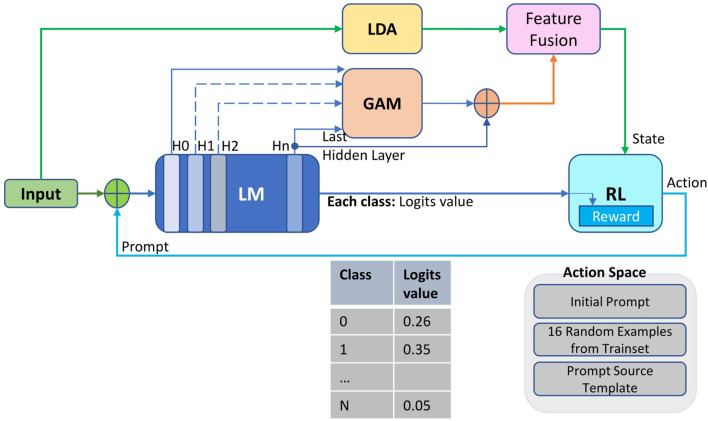
The LM-RL framework comprises three key components: LM for classification tasks, RL for prompt learning, and feature fusion outputs created by LDA and GAM integrating input data and different layer features from LM. These fusion outputs serve as state inputs for RL-based prompt learning, while LM’s classification logits provide reward. RL then generates prompt feedback to optimize the LM.

As illustrated in Figure 1, our network is built on the LM-RL framework. In this framework, the LM is employed for tasks such as classification, while the RL provides prompt feedback to the LM. The last hidden layer vectors of the LM, along with the corresponding logits values for each category in classification tasks, serve as the reward source for RL-based prompt learning. Additionally, the last hidden layer vectors, as well as vectors from other layers (H0, H1, H2), undergo further integration using global attention mechanism (GAM) and latent dirichlet allocation (LDA) for feature fusion. This fusion process yields a more informative RL state tailored for prompt optimization. Regarding the action space component in reinforcement learning (RL), similar to Tempera Zhang et al. (2023), the original prompts originated from Natural Instructions Wang et al. (2022), while the prompt templates were chosen from the Prompt Source Bach et al. (2022).

### 3.2 Fusion of multilayer features of language model

**FIGURE 2:**
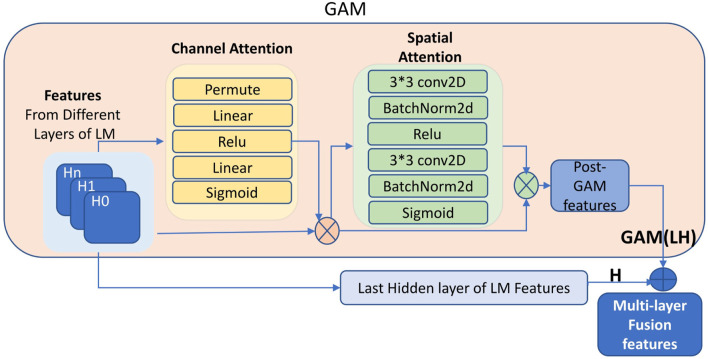
GAM fusion of features from multi-layers. GAM performs feature fusion across multiple layers of LM (H0, H1, Hn) to create GAM(LH). The final representation is formed by concatenating GAM(LH) with the LM’s last hidden layer output H.

Building on the insights from Section 2.2, which examines the use of various layers within BERT, our goal is to recombine features from different layers of the language model. Specifically, we aim to preserve the general semantic information captured by the lower pre-trained layers, while also retaining the task-specific features encoded in the higher layers closer to the output. To effectively leverage this multi-layer information, our approach consists of two main steps designed to explore and integrate features from different levels of the language model:

Firstly, as the network depth increases, valuable sparse features in the lower layers of LM may persist, particularly when dealing with small datasets. However, these features are often overshadowed or diluted in intermediate layers. To address this, we extract distinct hidden layers from the LM individually. We then leverage the strengths of the Global Attention Module Song et al. (2022), which operates on both spatial and channel dimensions, thus improving the understanding of sequence representations. By applying GAM to features from multiple hidden layers, we enable effective fusion of representations from these different layers. Specifically, features from three selected LM layers are treated as separate input channels to GAM. This design helps mitigate information loss in low-resource settings and strengthens the model’s ability to capture global contextual interactions. As indicated in Figure 2, in this context, the blue left inputs consist of features from different layers of LM (as shown in H0, H1, Hn). In contrast to the hierarchical sequence of layers in the language model, which is governed by the attention mechanism, our layer features operate in parallel, with the GAM enabling the exploration of interactions that span across these diverse layers.

Following the application of GAM to various layers, we extract additional features from these layers, which we refer to as ‘post-GAM’ features. Although GAM features originate from the lower and final layers, features derived from the lower layers may include valuable information because of their sparsity and specificity. However, these features might also introduce less valuable elements, such as noise, which can dilute the utility of the shared features in the original output of the language model’s final layer. To address this, instead of directly using the post-GAM features from different layers, we concatenate these features (referred to as GAM(LH) in Figure 2) with the output vectors from the language model’s last layer (H). For example, we extract features from both the first hidden layer (L) and the final hidden layer (H) of the language model (LM), each with a shape of [32, 1, 1024]. These two vectors are first added to form a fused representation, LH, which is then processed using the GAM, as illustrated in Figure 2, resulting in GAM(LH) (Shape: [32, 1, 1024]). The output is subsequently added to the final-layer features (H) of LM, resulting in the multi-layer fused representation GAM(LH)+H (Shape: [32, 1, 1024]). In the enhanced approach (GAM(LH)+H), both GAM(LH) and H are treated as equal contributors to the fusion process, this ensures that the common features of the last layer of the LM are reinforced. This strategy not only integrates additional information from the post-GAM, but also mitigates the risk of significant noise existing in the post-GAM features, which could otherwise dilute the impact of the common features from the language model’s last hidden layer. The concatenation here is intended to integrate richer feature information, and a simple addition operation is used, which introduces virtually no computational overhead. More complex methods for combining features from different layers could be applied here instead of simple addition; however, they would significantly increase computational overhead. As the goal of this study is to validate that further processing of features combined from different layers of the LM can provide more effective information. Therefore, prior to applying GAM to the multi-layer features—and before further enhancing the representation by integrating the post-GAM output with the H-layer—we adopt a simple addition of features as a lightweight fusion strategy, allowing us to validate the effectiveness of our proposed method. As such, more computationally intensive fusion methods are left for future work. The structure of this approach is illustrated in Figure 2.

### 3.3 Latent dirichlet allocation (LDA) fusion with LM

Considering the advantages discussed in Section 2.2 on the integration of LM and LDA, the results of LDA can serve as a foundation for various applications, such as document classification, similarity calculations and clustering. In this paper, the topics generated by LDA provide complementary information that differs from the features typically captured by LM tasks. To leverage this, LDA is employed as a feature extraction technique, enabling the integration of latent semantic features (topic features) into the output layer of the language model.

The implementation process primarily involves training the LDA model to generate topic outputs and integrating these features. To determine the optimal number of topics for the LDA model, we first train the LDA model on the training portion of each dataset. We then calculate the coherence of the topic, as described in Röder et al. (2015), which has been shown to correlate well with human judgment. In particular, Gensim provides several measures to evaluate topic coherence, enabling a more robust assessment of model performance.

**FIGURE 3:**
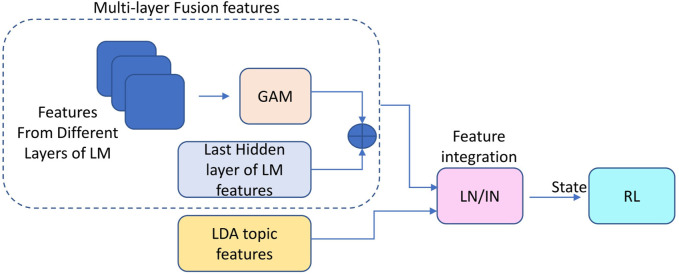
Multi-layer GAM fusion with LDA Integration. The multi-layer fusion features are combined with LDA topic features to form the final input for the RL network.

To enhance the integration of output from LM and LDA, it is essential to begin with feature normalization. Directly combining their outputs can lead to significant differences in scale, overgeneralization, and convergence challenges Peinelt et al. (2020). For semantic NLP tasks, merging LDA and LM output layers can be considered as the integration of distinct channels representing the same input. Layer normalization (LN) Lei Ba et al. (2016) is commonly recommended for such tasks. However, when working with small datasets, preserving the fine-grained details of both feature sets becomes critical. In such cases, instance normalization (IN) Ulyanov (2016) offers a better alternative. Given the limited data considered in this study, we opted for IN while also testing both LN and IN during the LDA topic fusion process in our experiments. This approach improves the integration of information between LDA and LM, resulting in substantial and valuable features for the subsequent RL network. As shown in Figure 3, the features of different layers of the LM are subjected to GAM. These are then combined with the last hidden layer’s features to produce multi-layer fusion features (e.g., GAM(LH)+H), which are further integrated with LDA topic features before being fed into the RL network. Specifically, due to the difference in shape between these two types of features (e.g., the former has a shape of [32, 1, 1024], while the latter is [1, 32]), the LDA features are first embedded using the language model (to obtain a shape of [32, 1024]). After reshaping both sets of features (to [1, 64, 1024]), they are concatenated to form the final fused representation. Then, Layer Normalization (LN) or Instance Normalization (IN) is applied to the fused representation. This combined feature, as illustrated in Figure 1, serves as the state for the RL network, preparing it for the subsequent prompt generation step.

## 4 Experiments

In this section, based on the LM-RL framework, we investigated two key aspects: first, the impact of incorporating specific language model layers into the GAM fusion, and second, the effects of adding LDA fusion to GAM. Subsequently, we compared the performance of our method with eight state-of-the-art baseline models. In Subsection 4.3, we conducted an additional experiment to evaluate how our approaches perform on a particular dataset with limited real-world data from the HRI scenario.

### 4.1 Datasets, baseline, and experiment setup

#### 4.1.1 Datasets

Our objective is to validate the effectiveness of our method across different datasets by evaluating few-shot text classification tasks. Our assessment includes sentiment analysis tasks on single-sentence datasets (SST-2 Socher et al. (2013), MR Pang and Lee (2005)) and multichoice datasets (AG_News Zhang et al. (2015)). Beyond semantic analysis, we also explored the method’s utility in a specific HRI scenario. We evaluated a dataset capturing interactions between behavior technicians (BT) and children with Autism Spectrum Disorder (ASD) during wh-question teaching sessions at a university-affiliated Applied Behavior Analysis (ABA) clinic. This task is unique, differing from standard classification or generation approaches due to its specialized context and limited dataset, as detailed in Section 4.3.

#### 4.1.2 Baseline

We evaluated the effectiveness of our methods by comparing them with a set of representative methods, which serve as the baseline. These methods include.

• Finetuning Devlin et al. (2019);

• Continuous prompt: Black-Box Tuning, AutoPrompt Shin et al. (2020); Devlin et al. (2019);

• Discrete prompt: Manual Prompt Bach et al. (2022), and In-Context Demonstration Min et al. (2022);

• RL prompt: RLPrompt Deng et al. (2022), Tempera Zhang et al. (2023).

#### 4.1.3 Experiment setup

We conducted text classification tasks using consistent configurations based on Tempera, ensuring a fair comparison. Our approach utilized RoBERTa-large as the language model and a Proximal Policy Optimization (PPO) reinforcement learning framework. The initial instructions came from Natural Instructions Wang et al. (2022), and the prompt templates were selected from PromptSource Bach et al. (2022). Each task used 16 randomly selected training samples per category, creating a small-sample dataset for prompt learning. We used the standard test set from the baseline methods for performance reporting. Regarding the topic generation part of LDA, we employed the LDA algorithm from the Gensim library to train and extract topics for each dataset, following an evaluation of topic coherence.

For the HRI dataset, we maintain consistent language and reinforcement learning models. However, the unique nature of the dataset and the extremely limited data risked overfitting with the standard 16-sample approach. To address this, we adapted our methodology by modifying input data format, PromptSource templates, and LDA topic design, as detailed in Section 4.3.

### 4.2 Experiments on few-shot learning classification tasks

#### 4.2.1 GAM on single layer experiments

In this section, we systematically investigated the performance implications of utilizing different hidden layers of LM for RL network input. Our preliminary exploration of the lower layers (L0, L1, L2) revealed minimal variability, leading us to focus on three critical layers: the first hidden layer (L, closest to input), the middle hidden layer (M, 12th layer in RoBERTa) and the final hidden layer (H, closest to model output). As illustrated in Table 1 in the single layer section, layers L and M demonstrated consistently lower performance compared to layer H. This performance disparity stems from the inherent information architecture of deep neural networks: Lower layers contain more redundant information, rendering them less effective for nuanced classification tasks.

**TABLE 1:** GAM on different layers (Accuracy %) of four datesets.

		SST-2	AG_News	MR	Clinic	Notes
Single Layer	L	59.1	65.1	80.7	21.9	Single layer L
	M	81.2	68.3	83.2	31.3	Single layer M
	H	**90.6**	**80.3**	**88.5**	**53.1**	Single layer H
	GAM(L)	74.2	68.7	83.5	28.1	GAM on single layer L
	GAM(M)	85.0	73.2	86.8	40.6	GAM on single layer M
	GAM(H)	83.8	77.9	88.4	46.9	GAM on single layer H
Two Layers	LH	64.6	80.0	86.9	37.5	Direct combination of two layers LH
	GAM(LH)	85.9	73.1	87.4	50.0	GAM-enhanced fusion of two layers LH
	GAM(LH)+H	**91.6**	**82.4**	**88.9**	**56.3**	Concatenating H with GAM(LH)

Bold indicates the highest accuracy result achieved on the dataset in that column.

Analysis of GAM Integration Across Network Layers: Our systematic evaluation of GAM application across different network layers revealed distinct performance patterns. When applied to the lower layer (L), which contains rich, unprocessed information, GAM demonstrated significant effectiveness, improving accuracy from 59.1 to 74.2 (as an example, on the SST-2 classification task). The middle layer (M) showed moderate improvement under GAM, with performance increasing from 81.2 to 85.0. However, applying GAM to the final layer (H) resulted in performance degradation from 90.6 to 83.8. This result stems from the final layer’s pre-existing feature refinement through the language model’s hierarchical processing; applying GAM creates a double-filtering effect that potentially obscures critical features. These findings suggest that GAM’s effectiveness is inversely proportional to the layer’s position in the network hierarchy, with optimal results achieved when applied to information-rich lower layers rather than pre-filtered higher layers.

#### 4.2.2 GAM on two layers and enhanced fusion experiments

Our investigation into multi-layer fusion revealed complex interactions between lower and higher layer representations. We evaluated two fusion approaches: direct combination of Lower and Higher layers (LH) and GAM-enhanced fusion with softmax (GAM(LH), in Table 1). The experimental results (LH = 64.6, GAM(LH) = 85.9) demonstrated performance inferior to the use of a single higher layer (H = 90.6). This unexpected outcome can be attributed to two key factors: first, the inherent noise in lower layer representations potentially degrading the refined features from the final layer, and second, the fusion mechanism’s implicit bias toward higher layer features. Despite the theoretical advantage of combining complementary information from multiple layers, the direct fusion approaches failed to effectively leverage the lower layer’s rich feature space while maintaining the higher layer’s discriminative power. These findings suggest that more sophisticated fusion strategies may be necessary to optimally combine multi-layer representations.

Subsequently, we improved the fusion methodology that leverages both GAM-processed layer combinations and pure higher-layer representations. This architecture, denoted as GAM(LH)+H in Table 1, implements a two-stage fusion process: first applying GAM to the combined lower and higher layers (GAM(LH)), then concatenating the original higher layer vector (H) with the GAM output. This enhanced approach achieved superior performance (GAM(LH)+H: 91.6) compared to previous configurations, suggesting the successful integration of complementary features across network depths. The performance improvement demonstrates that our architecture effectively preserves the higher layer’s refined features while incorporating valuable information extracted by GAM from the lower layer, resulting in a more robust and comprehensive feature representation.

#### 4.2.3 Fusion of LDA features experiments

We conducted a comprehensive analysis to optimize the Latent Dirichlet allocation (LDA) topic configuration, evaluating topic counts ranging from 10 to 200. Our selection criteria prioritized model parsimony while maintaining topic coherence, utilizing the leftmost inflection point on the coherence value smoothing curve as the optimal parameter. For example, in the case of the SST-2 dataset, we selected 32 topics as the optimal number, even though the highest coherence value is achieved at 35. In our experiments, we found that 30 to 50 topics worked well for all three datasets. After selecting the number of topics for each dataset, training was performed on different datasets to obtain their respective topic generation models. Since the results generated by LDA may contain a significant number of zeros, this could consume a substantial amount of memory. The results of LDA calculations were stored in the form of sparse matrices.

After topic features were generated, our experimental investigation of LDA feature incorporation followed a two-phase approach. Initially, we evaluated the direct integration of LDA-generated topic vectors with the final layer output of the language model (H + LDA in Table 2). Building on this foundation, we implemented an enhanced fusion architecture that combined our GAM-based layer fusion (GAM(LH)+H) with LDA topic features ((GAM(LH)+H)+LDA in Table 2). To account for the heterogeneous nature of data distributions and varying sample sizes, we implemented dual normalization strategies: Layer Normalization (LN) and Instance Normalization (IN). This comprehensive approach enabled systematic evaluation of LDA feature contributions while maintaining robustness across diverse data characteristics.

**TABLE 2:** Fusion of LDA (accuracy %).

	SST-2	AG_News	MR	Clinic	Notes
H	90.6	80.3	88.5	53.1	Single layer H
H + LDA	90.2	79.4	85.8	53.1	Direct integration of final layer with LDA
(GAM(LH)+H)+LDA: LN	91.8	84.6	88.7	56.3	GAM-based multi-layer fusion with LDA
(GAM(LH)+H)+LDA: IN	**93.1**	**86.2**	**89.3**	**59.4**	GAM-based multi-layer fusion with LDA

Bold indicates the highest accuracy result achieved on the dataset in that column.

#### 4.2.4 Comparison with the baseline

Our experimental results, presented in Table 3, demonstrate consistent performance improvements in all four datasets. Our method achieves accuracy gains over the best baseline values: 0.6% on SST-2, 0.7% on AG_News, 1.3% on MR, and 6.3% on Clinic. Evaluation metrics also include standard deviations calculated across multiple prompt sets (four distinct prompts for the clinic dataset and three for each of the remaining datasets). This systematic evaluation framework provides strong empirical evidence for the effectiveness of our approach in few-shot text classification tasks, while the inclusion of standard deviations offers insights into the method’s stability across different prompting strategies.

**TABLE 3:** Comparison with the baseline (Accuracy %). Evaluation on four datasets.

	SST-2	AG_News	MR	Clinic
Finetuning	80.6 (3.9)	84.9 (3.6)	67.4 (9.7)	43.8 (2.6)
AutoPrompt	75.0 (7.6)	65.7 (1.9)	62.0 (0.8)	40.6 (4.4)
Black-Box Tuning	89.1 (0.9)	93.2 (0.5)	86.6 (1.3)	-
Manual Prompt	82.8	76.9	80.9	53.1
In-Context Demo	85.9 (0.7)	74.9 (0.8)	80.6 (1.4)	40.6 (1.7)
RLPrompt	92.5 (0.8)	80.2 (0.7)	87.1 (0.4)	46.9 (2.3)
Tempera	91.9 (2.0)	85.5 (1.5)	88.0 (1.1)	46.9 (2.8)
Ours	**93.1(0.8)**	**86.2(1.0)**	**89.3(0.7)**	**59.4(1.8)**

Bold indicates the highest accuracy result achieved on the dataset in that column.

### 4.3 ABA clinic dataset experiment

Building upon our LM-RL methodology, we evaluated our approach on a specialized HRI dataset from therapeutic sessions at the ABA Clinic, where behavior technicians (BTs) interact with children during structured teaching tasks. The interaction typically begins with a wh-question from the BT, followed by the child’s response, which can trigger at least four types of BT responses: (1) positive reinforcement through social praise for a correct response, (2) error correction for incorrect answers, (3) prompts for no response, and (4) addressing other situations like a distracted or unresponsive child. This task presents unique challenges, combining both response assessment and strategic response generation, where BTs must evaluate the child’s response within the conversational context and formulate appropriate responses based on teaching objectives and historical progress. There are several challenges, including a very limited number of demonstrations available and inconsistencies in the decomposition of dialogue tasks. Humans rarely complete tasks in a single static step; even for the same task, conversations between BT and children can vary significantly. As a result, directly fine-tuning existing models or adding prompts—such as employing powerful language models for generative tasks—proves ineffective. Moreover, the closest pre-training dataset available Xie et al. (2021) focuses on causal relationships between sentence pairs, with topics largely centered around network blogs and photography-related encyclopedic content. This dataset has minimal relevance to the clinic context, where the objective is to generate the next sentence based on historical dialogue to advance the therapeutic session, rather than to address simple causal relationships.

In response to the absence of suitable pre-training datasets, we developed an approach inspired by the SWAG dataset architecture Zellers et al. (2018). In SWAG, a context (a question or a description) is given and the task is for the model to predict the most likely option from the four provided choices. To validate our approach, we reshaped the clinic data. Our methodology reformulates each dialogue instance as a concatenated sequence pair (‘current sentence +4 choices of next sentence’), enabling the language model to perform feature extraction and multi-class classification. Given the limited dataset size (118 training sentences and 32 test sentences), we implemented a hybrid prompt engineering approach combining SWAG-derived templates (‘appropriate continuation’, ‘how ends’, ‘first then’, ‘first then key’) with custom templates (‘next sentence’, ‘after this sentence’, ‘first then predict next’), while utilizing LDA features trained on all four datasets to enhance contextual understanding and model performance.

**FIGURE 4:**
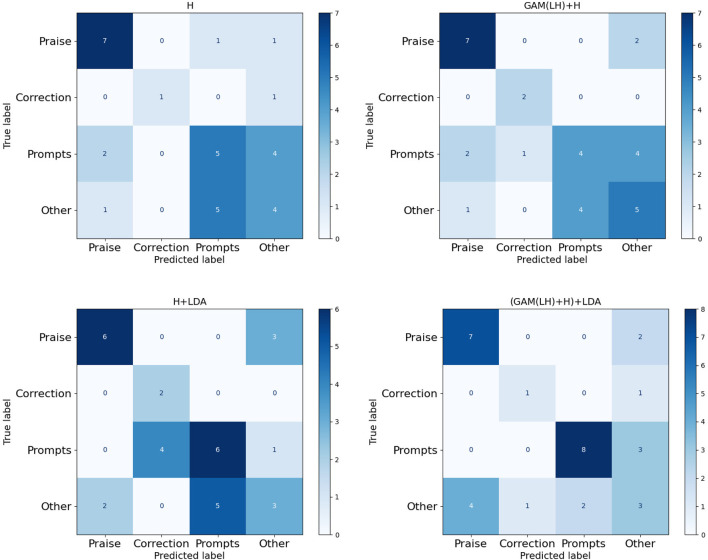
Confusion matrix of four fusion features (H, GAM(LH)+H, H + LDA, (GAM(LH)+H)+LDA). Single final layer (H), concatenating the H layer with the two layers (LH) GAM-enhanced fusion output (GAM(LH)+H), direct integration of final layer with LDA (H + LDA), GAM-based multi-layer fusion with LDA (GAM(LH)+H)+LDA). Four categories of BT’s responses in the clinic dataset: Praise, Correction, Prompts, Other.

**FIGURE 5:**
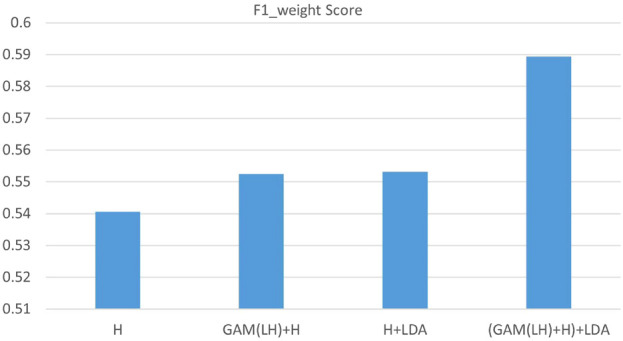
F1_weighted score of four fusion features of the clinic dataset.Single final layer (H), concatenating the H layer with the two layers (LH) GAM-enhanced fusion output (GAM(LH)+H), direct integration of final layer with LDA (H + LDA), GAM-based multi-layer fusion with LDA (GAM(LH)+H)+LDA).

**FIGURE 6:**
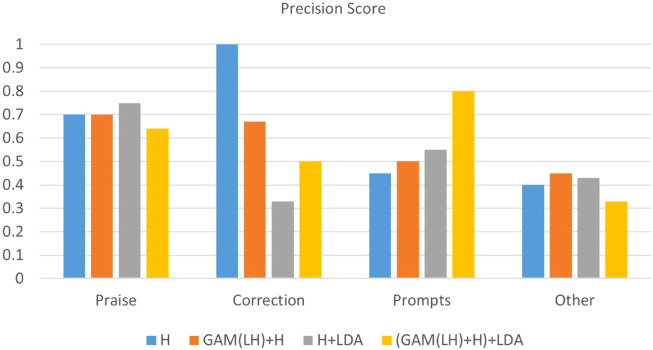
Precision score in four categories of BT’s responses in the clinic dataset: Praise, Correction, Prompts, Other.

The experiments were divided into a few parts: direct LM implementation (Finetuning), various prompt-based methods (AutoPrompt, Manual prompt, In-Context Demo), and LM-RL approaches (RLPrompt, Tempera, and our proposed LM-RL architecture). Initial LDA feature integration experiments utilizing topics generated solely from the clinic training dataset showed minimal improvement, attributed to limited data size and insufficient topic diversity. In such cases, what the model needs most is greater topic diversity, not just different topic modeling techniques applied to the same limited data. Simply switching models while keeping the dataset unchanged does not address the diversity issue. To address this, we conducted further experiments using LDA topics generated from larger, external datasets. Specifically, we applied the same LDA model to the combined content of the four datasets as the source for topic extraction. Since these datasets come from different domains, they do not introduce task-specific overlap with the clinic dataset, while still enriching the diversity of topic words. This study primarily aims to validate the effectiveness of incorporating LDA-derived topics. With access to more datasets, broader cross-dataset comparisons could be conducted in future work to further optimize topic selection. These results showed clear performance improvements, highlighting the effectiveness of supplementing low-resource datasets with richer topic representations for limited-data scenarios. Performance metrics, detailed in Tables 1–3 and illustrated in Figures 4–6, include confusion matrix analysis (accuracies: 0.541, 0.552, 0.553, 0.589), F1-weighted scores, and precision scores, providing comprehensive validation of our approach.

## 5 Discussion

Our GAM(LH)+H configuration demonstrates superior performance in few-shot learning scenarios (Table 1), treating the H-layer vector and GAM(LH) output as equal contributors. This approach enables the capture of valuable features that might be overlooked in traditional hierarchical processing, particularly beneficial for prompt learning in few-shot scenarios.

Further analysis of feature integration (Table 2) reveals that while direct H + LDA fusion showed minimal improvement over baseline, our GAM-enhanced architecture with Instance Normalization fusion (G (LH)+H)+LDA achieved significant gains. This architecture successfully incorporates features beyond the representations of the standard language model, as evidenced by the comprehensive metrics. Combining the results from Tables 1, 2, we observe that the output from the final layer alone (H: 90.6) serves as a strong baseline. The enhanced approach (GAM(LH)+H) achieves superior performance (91.6 in Table 2), demonstrating the benefit of integrating features from multiple layers using the GAM mechanism. In contrast, directly combining LDA-generated topic vectors with the final layer output (H + LDA, 90.2 in Table 2) does not surpass the performance of using the final layer alone. However, when we implemented an advanced fusion architecture that combines GAM-based layer fusion with LDA topic features ((GAM(LH)+H)+LDA in Table 2), further improvements were achieved. This is because both modules contribute complementary strengths: GAM enhances the capture of hierarchical information from the language model, while LDA provides topic-level semantic cues. Their complementarity enables more informative features to be incorporated into training, leading to improved overall performance.

The analysis of the ABA clinic dataset (Figures 4–6) shows consistent improvements in accuracy and F1_weighted scores, particularly in the ‘Prompts’ category classification. The confusion matrix (Figure 4) reveals that the model performs well in the ‘Praise’ and ‘Prompt’ categories when using GAM-based multi-layer fusion with LDA. However, the ‘Correction’ and ‘Other’ categories exhibit noticeable misclassifications. The F1-weighted scores reinforce this trend, with GAM-based multi-layer fusion with LDA achieving the highest scores, indicating a good balance between precision and recall in ‘Praise’ and ‘Prompt’. In contrast, precision analysis shows that ‘Praise’ and ‘Prompt’ benefit from higher precision, while ‘Correction’ and ‘Other’ have significantly lower precision. Category-specific performance varied: ‘Praise’ and ‘Prompts’ maintained strong baseline performance, while ‘Correlation’ remained limited by sample size, with fewer training samples compared to the other two categories. And ‘Other’ faced training data complexity challenges due to the complexity of the training data. In this experiment, all data that did not belong to the three categories were classified as ‘Other’, resulting in a highly complex ‘Other’ category. This category essentially consisted of a combination of various types, making it difficult for the model to classify accurately.

**FIGURE 7:**
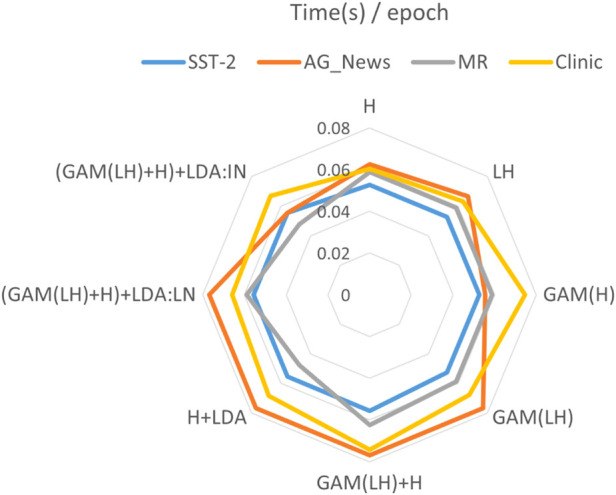
Time(s) of each epoch in four datasets. Single final layer (H), two layers (LH), GAM-enhanced final layer (G(H)), GAM-enhanced two layers (G (LH)), concatenating the H layer with the two layers GAM-enhanced fusion output (GAM(LH)+H), direct integration of final layer with LDA (H + LDA), GAM-based multi-layer fusion with LDA (GAM(LH)+H)+LDA), dual normalization strategies: LN and IN.

All LM-RL-based methods outperform non-LM-RL baselines, with our approach achieving higher accuracy and lower standard deviations (Table 3). Although Black-Box Tuning shows superior performance on ‘AG-News’ 4-class classification, our method consistently outperforms prompting baselines. As shown in Figure 7, the training speeds (measured in seconds per epoch) of different methods across four datasets are presented. Due to the relatively small size of the Clinic dataset, its values were normalized to maintain consistency in scale within the chart. It can be observed that applying GAM to different layers does increase computational cost. However, the subsequent inclusion of H-layer features does not expand the length of the feature vector, and the number of topics (32) used in the added LDA features is relatively small. These additions do not significantly impact feature dimensionality or training speed. The training speeds of various methods are fairly comparable on the SST-2, MR, and Clinic datasets. The two notable exceptions occur on the AG_news dataset, where the training times for (GAM(LH)+H)+LDA with LN and IN differ more noticeably. We believe this is due to the text features in AG_news being more sensitive to these normalization techniques, highlighting the importance of deeper feature exploration tailored to different types of data.

## 6 Conclusion and future work

We present a novel framework integrating GAM with multi-layer features and LDA topic incorporation, demonstrating enhanced feature extraction in few-shot learning contexts. Our approach’s effectiveness stems from strategic multi-layer feature integration via GAM and feature space enrichment through LDA topics; the robust performance across diverse datasets validates our approach’s versatility and effectiveness in optimizing prompt generation for few-shot learning applications.

Current limitations include the dependency on layer-specific feature vectors, potentially constraining applicability to diverse language model architectures. Future work will explore advanced LDA metrics, enhanced topic generation with other models, and expanded multi-layer information strategies. Automation of task-specific feature selection for prompt optimization remains a critical challenge, particularly for human intent, environmental context, and engagement modeling.

The ABA clinic dataset analysis reveals methodological constraints in transforming therapeutic interactions into multiple choice format, primarily in the ‘Other’ category classification. Future developments will focus on granular data categorization and fine-tuned generation models with ABA therapy scripts, aiming to better capture therapeutic interaction complexity while maintaining computational efficiency.

## Data Availability

The raw data supporting the conclusions of this article will be made available by the authors, without undue reservation.
